# Moving online: young people and parents’ experiences of adolescent eating disorder day programme treatment during the COVID-19 pandemic

**DOI:** 10.1186/s40337-021-00418-4

**Published:** 2021-05-24

**Authors:** Phillipa Louise Brothwood, Julian Baudinet, Catherine S. Stewart, Mima Simic

**Affiliations:** 1 Maudsley Centre for Child and Adolescent Eating Disorders (MCCAED), Maudsley Hospital, De Crespigny Park, Denmark Hill, London, SE5 8AZ UK; 2 Department of Psychological Medicine, Institute of Psychiatry, Psychology and Neuroscience, King’s College London, De Crespigny Park, Denmark Hill, London, SE5 8AZ UK

**Keywords:** Online therapy, Anorexia nervosa, Adolescents, Day programme, Partial hospitalisation program (PHP), Covid-19 pandemic

## Abstract

**Background:**

This study examined the experiences of young people and their parents who attended an intensive day treatment programme for eating disorders online during the global COVID-19 pandemic.

**Methods:**

Online questionnaires were completed by 14 adolescents (12–18 years) and their parents (*n* = 19). The questionnaires included a mixture of rating questions (Likert scale) and free text responses. Free text responses were analysed using reflexive thematic analysis.

**Results:**

Three main themes were identified: 1) New discoveries, 2) Lost in translation and 3) The best of a bad situation. This study provides insight into the benefits and pitfalls of online treatment delivery in the adolescent day programme context, which has rapidly had to become part of the everyday therapeutic practice. Results indicate that there are advantages and disadvantages to this, and that parents and young people’s views differed.

**Conclusions:**

This study suggests that the increased accessibility provided by online working does not necessarily translate to increased connection. Given the importance of therapeutic alliance in treatment outcomes, this will be an important consideration for future developments of online intensive treatments.

## Plain English summary

This study examined the experiences of young people and their parents who attended an intensive day treatment programme for anorexia online during the COVID-19 pandemic. Online questionnaires were completed by 14 patients aged 12–18 years and 19 of their parents. The questionnaires included a mixture of rating questions (for example: How would you rate your experience with online therapy from 1 to 10?) and free text responses. The authors explored the free text responses and identified three common themes. These were 1) New discoveries- for the parents and young people, online therapy was a new experience, 2) Lost in translation- working online meant some aspects of communication were lost, 3) The best of a bad situation- online therapy was better than no therapy at all. This study provides insight into the benefits and pitfalls of online therapy in a day programme setting. Specifically, this study suggests that the increased accessibility provided by online working, does not necessarily translate to increased connection. Given the importance of building a positive and trusting relationship between the young person, their family and their therapist, this will be an important consideration when thinking about future development of online treatment programmes.

## Background

It is now well established that, for a significant minority of young people, first line eating disorder treatments are not having the intended effects [1]. Full remission rates at the end of treatment remain modest for both anorexia nervosa (20–50%) and bulimia nervosa (~ 40%) [2]. Day programme (DP) treatment is becoming an increasingly popular alternative to inpatient care for this group of vulnerable young people. DP treatment leads to improvements in physical and psychological factors associated with eating disorders [3].

A recent randomized controlled trial has demonstrated the non-inferiority and cost-effectiveness of DPs compared to inpatient treatment [4]. Given the high cost of inpatient treatment [5, 6], disputed outcomes beyond medical stabilisation [7–9], and longer-term (2.5 year) outcomes favouring DP over inpatient treatment in regard to body mass index, relapse and readmission rates, there is a need to continue developing DP treatment models.

The Intensive Treatment Programme (ITP) is a day programme at the Maudsley Centre for Child and Adolescent Eating Disorders, London, UK. ITP admits young people and their families who have either not responded to outpatient treatment or who require support stepping down from inpatient care. The aim of ITP is to establish a trajectory towards recovery and re-engagement with outpatient treatment, not full remission. ITP has been operating since 2010 and is effective at supporting adolescents to improve their physical health, eating disorder psychopathology and mood [10], as well as a range of social and personality features associated with bio-temperamental overcontrol [11].

In response to the national lockdown in March 2020 due to the COVID-19 pandemic, all non-urgent face-to-face patient contact ceased. All but essential, crisis appointments moved quickly to an online platform. To ensure continuity of care, all aspects of the ITP were quickly replicated and restructured for online delivery. Dependent on the evolving national COVID-19 restrictions, the service has been run virtually, or as a hybrid of part virtually part face-to-face, since March 2020.

While several studies have emerged detailing guidelines and the experience of moving outpatient eating disorder treatment online [12, 13], no study to date has explored this in an adolescent day programme context. Emerging evidence suggests it has been a promising way of working for adults with eating disorders during the pandemic [14, 15]. The aim of this study was to understand the experience of participating in ITP online during the COVID-19 pandemic from the perspective of the young people and their parents.

## Methods

### Participants

A total of 28 families (28 young people and 51 parents) participated in ITP between March and November 2020. During this period ITP was either fully or partly virtual. Four young people were identified by the multidisciplinary team as too vulnerable to participate in this study due to either physical or psychiatric risk. The remaining 75 individuals were invited to participate. All participants received the survey via email with an explanation of its purpose, assurance of confidentiality and that it was optional, followed by two subsequent reminders.

A total of 14 young people and 19 parents completed the survey (58 and 37% of those contacted, respectively). All adolescents were between 12 and 18 years of age and met DSM-V criteria for anorexia nervosa, restrictive subtype. One identified as male, the remainder as female. No further demographic information was collected to ensure confidentiality.

### Treatment context and adaptations for online working

Prior to the COVID-19 pandemic ITP was operating 5 days per week (Monday to Friday). Approximately 6 h of face-to-face contact was offered per day with a capacity for eight to twelve families at any one time. The program treatment model is primarily based on the principles of the family therapy for anorexia nervosa (FT-AN) [16], in which both the young person and their family play a central role in the process of recovery. Alongside weekly FT-AN, other interventions include supervised meals, group therapy, dietetic reviews, medication reviews and weekly individual therapy. The multidisciplinary team comprises psychiatry, paediatrics, psychology, nursing, family therapy, art therapy, and dietetics. ITP programme details and outcomes have been published elsewhere [10, 11].

In response to government restrictions on face-to-face working due to the COVID-19 pandemic, all aspects of ITP were adapted for online working, where possible. This included moving individual and family therapy, young person and parent groups, meal support and education support online. Multi-family meal support was the only element of the programme that stopped during the COVID-19 pandemic.

Individual, family and group therapeutic content followed the same structure and timings as the face-to-face programme. Weighing happened at home with a parent present either just before, or at the beginning of sessions. The young people continued to receive daily therapeutic groups including, radically open dialectical behaviour therapy, cognitive behavioural therapy, cognitive remediation therapy, food and nutrition, art therapy and yoga therapy groups. Parents attended a weekly parent skills group. Group materials, role-plays and small group exercises were all adapted to fit the online platform, using online worksheets, polls, online video clips and breakout room exercises.

Daily meal support was also moved online. Everyone attending ITP was offered support with at least one meal daily (Monday to Friday). One clinician would be present virtually and at least one parent was required to be with the young person during the meal. Coaching was provided as needed, as were encouragements, distractions and reminders of goals. Once the meal was finished, one person also led a game to model the use of distraction after meals. Meal timings remained the same as during face-to-face ITP. In the event of meal non-completion, parents were encouraged to persist, and if needed employ strategies and problem solve together with the clinician. A clinician could then specify a time to re-join or check in with the family later to ensure completion, if needed.

Importantly, the program was never completely virtual. Urgent and crisis appointments continued to be offered throughout the study period. These were offered in the event of increased physical risk (e.g., rapid weight loss, meal refusal) or psychiatric risk. These face-to-face appointments functioned to manage risk only, with the aim of returning to online working as soon as possible.

Once lockdown restrictions eased in July 2020, the programme also moved to a more hybrid model of service delivery with a smaller number of participants and social distancing in place. Two days remained completely online (Tuesday and Friday). The remaining three (Monday, Wednesday and Thursday) could be accessed either face-to-face or online. Face-to-face attendance was prioritised for those presenting with highest risk, with the expectation that the young people would transition to online working as soon as possible. One these days, two meals and one group were offered face-to-face with social distancing in place. The group could be attended both in person and online (for those fully virtual). Online meal support continued to be offered as described above for those not attending ITP in person. All other aspects of the programme (education, individual therapy, family therapy, parent group, medical appointments and dietetic reviews) remained online.

### Data collection

Two questionnaires, one for young people and one for their parents, were created to understand the experience of attending ITP online using the Qualtrics XM platform. This platform was chosen as it provided a quick, accessible and confidential method for participants to respond, either on their computer or mobile phone. This allowed for increased accessibility and confidentiality [17], which was felt to be key in encouraging participation and openness. The length of the survey was also considered for the former reason. A mixture of quantitative rating questions (Likert scales) and qualitative free text questions were used to generate the data. Free-text questions were interspersed with Likert scale questions to allow for expansion on specific items as well as allowing views about global experience.

Rating questions captured data on the overall experience of ITP (1 = poor, 10 = excellent), whether working online impacted treatment quality (1 = not at all, 10 = a lot), how comfortable participants felt in online treatment (not comfortable, quite comfortable, very comfortable) and whether technology impacted treatment experience (1 = not at all, 10 = a lot).

Participants also rated their experience of specific treatment components online, including meal support, individual and family sessions, group treatment, medication reviews and dietetic reviews. Participants were asked to rate how helpful they found each component (not at all helpful, somewhat helpful, very helpful) and how it compared to face-to-face working, if they had experienced both (less helpful, the same, more helpful). Lastly, participants were asked about their preference for treatment once restrictions eased (online, face-to-face, don’t mind).

Free text questions specifically asked about the experience of online therapy and for further detail surrounding responses to rating scales. The data were stored automatically on the Qualtrics XM platform as the participants responded and the responses could be viewed per question or per participant.

The authors critically examined their own role and influence in formulation of the research question and survey and aimed to mitigate any potential bias by having the survey reviewed by other members of the ITP team prior to sending it to participants.

This study was approved as a service evaluation project by the South London and Maudsley NHS Foundation Trust National and Specialist Child and Adolescent Mental Health Service Evaluation and Audit Committee.

### Data analysis

A summary approach was used for quantitative data. Percentages, medians and interquartile ranges (IQR) were calculated for responses to the rating questions, where applicable. Free text data were analysed using Reflexive Thematic Analysis [17–19] within a critical realist framework, which views meaning and experience as subjective and influenced by social and cultural context. An inductive and semantic approach was identified as appropriate for the data set. This allowed two authors (PLB and JB) to be guided and directed by the explicit content of the data rather than looking at existing concepts or assumptions or making inferences as in other approaches [20]. This felt most informative as online therapy is a relatively new phenomenon. The six phases of thematic analysis as outlined by Braun and Clark [18] were followed. Both authors (PLB and JB) independently spent time ‘dwelling with’ the data [18] to become immersed and familiar with the content. Codes and then themes were then generated independently before the authors met to discuss findings. Initially, young people’s and parents’ responses were analysed separately with separate themes developing. However, through the process of discussion, the themes were revisited, reviewed and refined and it was felt that broader themes encompassing both data sets best reflected the raw data. A final consensus was then reached with no difficulties noted as the themes identified individually mapped easily onto each other.

## Results

### Quantitative data

Generally, the experience of online ITP was rated positively. Parents rated it slightly higher (median = 8/10, IQR = 6.5–10) than young people (median = 6.5/10, IQR = 5–7). However, participants said the quality of treatment was partly impacted due to moving online (young people: median = 5.5/10, IQR = 4–6.75; parents: median = 5/10, IQR = 2.5–8). Parents felt more comfortable in online groups than young people. Seven (37%) parents reported feeling ‘very comfortable’, whereas none of the young people did. Most participants reported that issues with technology impacted the experience of treatment, with young people reporting more of an impact (median = 5, IQR = 5–8) compared to parents (median = 2, IQR = 1–5). Despite these difficulties, ten (71%) young people and 15 (79%) parents felt that the programme could not have done anything differently to better meet their needs.

With regard to specific program components, each component was rated as somewhat or very helpful by at least 71% of young people and 89% of parents. Although, two young people (14%) reporting that virtual meal support was not helpful at all and two (14%) did not find individual sessions helpful online. Also, four young people (29%) and two parents (11%) did not find online dietetic support helpful.

When online working was compared to face-to-face support responses were more varied (see Table 1). Young people generally found all online components of treatment less helpful than their parents. Specifically, meal support, family sessions and individual sessions were all rated as less helpful online than in-person by the majority (all > 66%) of young people. Medication and dietetic reviews were an exception, both of which were considered to be similarly helpful online and face-to-face by most. The majority of treatment components were rated as being similar online as face-to-face by parents. Regarding group treatment, only four young people had the experience of both online and virtual formats. All four (100%) reported online group treatment to be less helpful. In comparison, five (50%) of the parents experienced online parent group as more helpful than face-to-face.

**Table 1:** Young person and parents’ comparison of online and face-to-face treatment components

	***n***		Less helpful than face-to-face		The same as face-to-face		More helpful than face-to-face	
	***YP***	***P***	***YP***	***P***	***YP***	***P***	***YP***	***P***
Family sessions	12	12	8 (66.7%)	1 (8%)	2 (16.7%)	11 (92%)	2 (16.7%)	0 (0%)
Individual sessions	12	n/a	8 (67%)	n/a	3 (25%)	n/a	1 (8%)	n/a
Meal support	10	9	7 (70%)	2 (22%)	3 (30%)	5 (56%)	0 (0%)	2 (22%)
Groups	4	10	4 (100%)	1 (10%)	0 (0%)	4 (40%)	0 (0%)	5 (50%)
Medication reviews	10	10	3 (30%)	1 (10%)	7 (70%)	8 (80%)	0 (0%)	1 (10%)
Dietician reviews	9	8	4 (44%)	1 (12.5%)	5 (56%)	7 (87.5%)	0 (0%)	0 (0%)

*Abbreviations*: *P* Parents, *YP* Young person

Regarding preferences moving forward, the data was quite varied (see Table 2). For young people, there was a preference for returning to face-to-face support, particularly for meals, family sessions, individual sessions and group treatment. This was not the case for medication reviews or dietetic appointments with more than half the young people (> 50%) either not minding or preferring virtual appointments. Conversely, most parents (> 60%) did not mind or preferred virtual support moving forward for all treatment components.

**Table 2:** Preferences for treatment format once face-to-face working is available again

	n		Face-to-face		Online		Don’t mind	
	***YP***	***P***	***YP***	***P***	***YP***	***P***	***YP***	***P***
Family sessions	13	16	8 (62%)	5 (31%)	0 (0%)	7 (44%)	5 (38%)	4 (25%)
Individual sessions	13	16	9 (69%)	n/a	0 (0%)	n/a	4 (31%)	n/a
Meal support	13	16	8 (62%)	6 (38%)	2 (15%)	6 (38%)	3 (23%)	4 (25%)
Groups	13	16	10 (77%)	2 (13%)	0 (0%)	11 (69%)	3 (23%)	3 (19%)
Medication reviews	13	16	4 (31%)	3 (19%)	2 (15%)	9 (56%)	7 (54%)	4 (25%)
Dietician reviews	13	16	6 (46%)	2 (13%)	0 (0%)	9 (56%)	7 (54%)	5 (31%)

*Abbreviations*: *P* Parents, *YP* Young person

### Qualitative responses

**Fig. 1:**
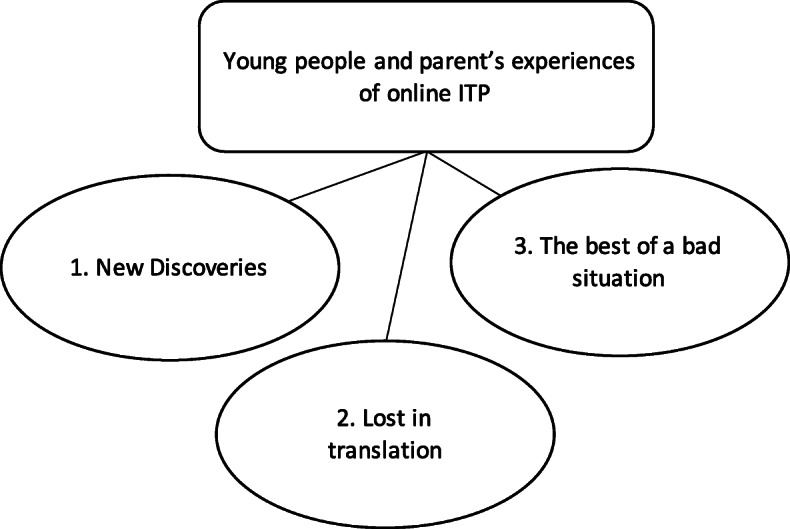
Main themes of young people and parents’ experiences

Three main themes were identified following analysis of free text responses (see Fig. 1). These were 1) New discoveries 2) Lost in translation 3) The best of a bad situation. These themes are described further below and illustrated with relevant quotations. Quotes are as written, with only minor adjustments to spelling to aid ease of reading.

#### New discoveries

Both parents and young people found that participating in the online programme allowed for a new discovery; increased flexibility and accessibility, which they had not had when face-to-face.

Parents found the increased accessibility afforded by online work helpful in terms of flexibility, allowing work schedules and family life to continue as normally as possible. Similarly, the online format reduced the burden of travel time and expenses, which is a significant benefit for national referrals and those living outside of London. One parent commented that they would like the online element to continue and many requested/suggested a hybrid model of service delivery in the future.

The benefits of increased accessibility and flexibility were also noted by the young people who said that they were able to access treatment from different places, such as home, school and work. Some young people commented that it felt more comfortable being at home and that it was less of a time commitment. For parents, similar views were expressed. Online contact allowed for a level of availability and support that was not present before. An important finding was that although this was noted as a benefit no young people commented on wanting the online work to continue.

*Parent: “Overall found it far more flexible and able to maintain life with our other children”*

*Young person: “[You were] able to be more flexible with time and date of appointments and being able to do it from anywhere”*

*Parent: “Was more immediate if we needed emergency support”*

Several parents and young people particularly commented on how increased accessibility improved meal support. Five parents specifically noted how much they appreciated the advice offered online. They said this helped connect what was discussed in theory in sessions to the practical  task of meal supervision with an ITP clinician. Online meal support at school was also an identified benefit. For those experimenting with independent eating, virtual check-ins, planning and meal support at school added increased support, whilst minimising disruption of education. Similarly, one young person commented that being at home made the experience more translatable to their everyday life.

*Parent: “Meal support online effectively coached us as parents to do meal support ourselves more effectively”*

*Young person: “Having meal support at home got me used to eating in my own house”*

This connected with a more general idea that increased accessibility allowed for better translation of content to the ‘real world’. Despite these benefits, it is worth noting that most young people (70%) and a few parents (22%) rated online meal support less helpful than face-to-face meals on Likert scale responses (see Table 2).

Increased accessibility was also associated with increased attendance and productivity for some. Six parents noted that online working meant all family members were able to attend sessions more frequently. This was particularly helpful in families where the parents are separated.

*Parent: “[the format promoted] attendance of both parents”*

*Parent: “As we are two sets of parents the virtual sessions have allowed us to actually discuss [our daughter] together”*

Two young people also discovered that sometimes the online sessions were more productive than face to face. They noted that it was easier to listen, and that being online made it easier to write and listen simultaneously.

#### Lost in translation

A predominant theme, generated from all participants, was based around human connection. Online work meant that there were elements that were somehow ‘lost in translation’. Accessibility did not equal connection and there were disruptions observed in the therapeutic alliance.

Many of the comments made by the young people about their experience touched upon it feeling less personable via a screen. For many it was almost a ‘barrier’ to forming connections with both their therapists and the multi-disciplinary team as a whole. This was echoed by parents too.

*Young person: “ [It is] harder to be open when it’s virtual”*

*Parent: “[It was] a little less human”*

Four parents noted that their child did not like engaging with the screen. One stated that this, at times, could undermine the support offered and was a barrier to the development of a strong therapeutic connection. The screen as an emotional barrier was also something parents experienced. However, two parents noted that ‘barrier’ was sometimes an aid for the young people. Overall though, there was a discrepancy between parents and young people.

*Parent: “The family sessions have worked particularly well online: our daughter seems to open up more”*

*Parent: “I have found it incredibly helpful, however our daughter hates virtual support”*

Online working seemed to necessitate adjusting to new social cues and ways of reading social and emotional responses. One young person noted that it was uncomfortable needing to mute/unmute yourself to participate. Others commented that it was harder to read emotions and they felt like there was more of a pressure to speak. When asked about the parent group, parents answers reflected those of the young people in terms of navigating a new communication tool.

*Parent: “It can be stilted - sometimes you want to say something but someone else is talking”*

*Parent: “a raised hand function would be helpful”*

The more traditional ways that relationships are built were noted to be absent in the online format. This was evident for parents and young people alike, particularly when reflecting on groupwork. These included people feeling less bonded, missing opportunities for physical contact (e.g., a parent offering a hug to another parent), talking during breaks, etc. Alternative ways of connecting were suggested by some, such as a parent chat group separate to the treating team.

*Parent: “Also, inevitably, you don't bond so much with other parents (we did an in-person parent group in the community, and got to know the others better)”*

*Young person: “you don't really know the people very well because you don't get to talk to them outside of group time so it can be harder to open up/give personal examples”*

This also highlighted the value of the environment surrounding the therapy sessions. One young person noted that there was not the space to get into the therapeutic mindset. A parent also wrote that their daughter missed the social contact with other young people that came from face-to-face attendance, which can also be part of the therapeutic process.

Intangible human connection was interrupted by tangible technical difficulties on occasion. Nine of the fourteen young people noted that technical difficulties interrupted the therapeutic experience.

*Young person: “Sometimes when the WIFI glitches or someone freezes it can be difficult if you are in the middle of talking about something important”*

*P: “Sometimes the connection cuts so it’s hard to keep up with what’s going on”*

Several parents and young people noted either the benefits of having met the therapist/team before moving online, or a desire to have done so. Some people suggested this might help build and maintain the therapeutic alliance.

*Parent: “I think if the relationship can be established in person first, then online has worked fine for our daughter”*

*Young person: “I would’ve struggled with doing the whole ITP programme online”*

When asked how the programme could improve this, young people said we need to think of ways to make meetings feel more personal and get creative with how to connect more.

#### The best of a bad situation

Online therapy at ITP was a response to the global COVID-19 pandemic with an aim to provide some level of continuity of care in an unprecedented situation. It was not a planned trial of a new method of delivering therapy. This was reflected in many of the responses, as was the gratitude of the participants for the continuity of care during a very difficult time.

*Young person: “I think it’s been as good as it could've been given the circumstances”*

*Parent: “it's better than sitting wearing a mask and not seeing facial expressions!”*

Most valued remaining connected to others in some capacity, however stilted it felt at times. While online working was more comfortable, accessible and less stressful for some, generally when compared to face-to-face working, it was thought to be equivalent or not quite as good. Importantly, four young people did not feel there was anything helpful about online therapy.

*Young person: “it kept the therapy going but didn’t make any improvements”*

## Discussion

This study aimed to investigate young people and their parents’ experience of participating in intensive day programme treatment online during the COVID-19 pandemic. Generally, young people and parents found virtual ITP helpful and more accessible, although they noted they felt slightly less comfortable at times. They also found going virtual did impact the quality of treatment. There was generally a preference from the young people for most ITP interventions to return to face-to-face working when possible, with the exception of medication reviews and dietetic appointments. Parents generally stated a preference for keeping meetings virtual. This matches the experience of adolescents, carers and clinicians in the outpatient treatment context during the pandemic [12].

The qualitative analysis from a critical realist approach also highlighted that traditional day programme treatment differed to online working in some fundamental ways. When face-to-face, the timings of appointments and structure were described as more fixed and rigid and the conversation more fluid. Conversely, during online therapy the opposite was described. The timings and practical elements of treatment, such as requesting an appointment, became more flexible, whereas the relational element became more rigid and stilted with the introduction of a screen. The screen acted as both a literal and also somewhat intangible barrier which disrupted the therapeutic connection for many. Although, it is worth noting, a few young people found the ‘distance’ helpful when trying to open up and talk about their experiences.

A surprising element was that young people found online therapy more challenging than their parents, who appreciated the flexibility and compatibility with family life more. Parents commented that they had used Microsoft Teams for work, which may in part explain why they felt more comfortable, although many young people were also using the same platforms to attend school. This was unexpected as a huge part of communication for today’s young people is via online platforms [21] and 50% of 10-year-olds own their own smartphone [22].

Increased availability and accessibility of the treating team does not necessarily equal increased connection, engagement or therapeutic alliance. This highlights the need to find new ways of building trusting relationships and a deeper connection online if virtual working is to persist so prolifically beyond the pandemic. Human connection was the predominant theme for all participants. The results suggest that intimacy and trust are partly built and reinforced during day program treatment in the moments ‘between therapy’ (e.g., moving the chairs around and standing in the corridor waiting for the session and making small talk). Online work essentially removes these ‘extras’ and can make the sessions more content-driven, with potentially important social interactions lost.

A broader question raised by the current study is whether the therapeutic alliance and engagement created during online ITP is sufficient. Therapeutic alliance is associated with improved outcomes in child and adolescent psychotherapy generally [23], as well as in eating disorder focused family therapy specifically [24], suggesting it may be important in eating disorder DP treatment. However, the same relationship between alliance and outcome does not necessarily exist for online treatments. Emerging data suggests that therapeutic alliance is not necessarily associated with improved treatment outcomes, rather it is agreement on treatment goals and tasks that appears crucial when working online [25].

Despite the reported equivalence in alliance ratings between online and face-to-face treatments [25], this has not been reported in a recent meta-analysis [26], nor indicated in the current study. The current findings are similar to those of a recent investigation of online treatment experience during the covid-19 pandemic in the outpatient context [12]. There was a strong preference, particularly by the young people, for a return to face-to-face working. This may, in part, be due to study design. All families had had some experience of face-to-face treatment, whether it was in ITP or from the service they were referred from, and were asked to directly compare both formats. If families only had the experience of online treatment, satisfaction with treatment may have been different.

Discrepancies also arose depending on the way questions were presented. Even though none of young people, and only a minority of parents, rated a preference for interventions to continue online into the future on Likert scale questions, both groups noted benefits for several types of interventions in free-text responses (e.g., meals, dietetic support, medication reviews). These discrepancies provide richness to our understanding of the experience and can be seen as complimentary, rather than opposing [26]. Even if face-to-face working is ultimately preferred, the benefits of in-home, flexible and accessible support might be useful to consider in future treatment development, regardless of format.

An additional factor to consider is the unique impact of the pandemic. Families were asked to compare online and face-to-face working at a time when face-to-face interaction was severely restricted due to government enforced lockdowns. Even as clinicians, the authors noted a strong desire for human interaction outside of the virtual setting. Perhaps if the two treatment formats were not so directly compared and families had been asked at another time when social interaction was much more available, the pattern of responding would have been different. Nevertheless, outside of the pandemic setting, people with restrictive eating disorders do report experiencing distant relationships and high levels of loneliness [27], suggesting the therapeutic relationship may be a particularly important consideration in this group.

Given the many benefits of online working, the differences reported in engagement may also be worth tolerating. Whether it impacts upon outcome is yet to be determined in this setting and for eating disorder treatment specifically. In other settings, online and face-to-face interventions are reported to have similar outcomes [28], although it has been noted that some face-to-face work may be needed to compliment online working for it be effective for eating disorder symptom improvement [29]. Within the current study, one element that did help to promote the therapeutic relationship was meeting the ITP team initially face-to-face. This suggests that a hybrid model may be the most appropriate way forward.

Another important finding was the new social etiquette of online working. As has been reported in several other studies, communication in online group-based interventions can be stilted and may require structure and protocols to optimize it [28]. It remains to be seen whether with time and as online video communication becomes more embedded into our society, the communication becomes more fluid and less stilted.

Lastly, no young people mentioned any challenges associated with seeing themselves on the screen. This was anticipated to be a potential difficulty of using an online format as body image distortion is a core diagnostic feature of anorexia nervosa [30]. Furthermore, no young people and only one parent, described finding eating on a screen challenging. This may be due to young people feeling uncomfortable to report this or it may not have been a primary concern for them.

### Study strengths and limitations

This study had several strengths. The combination of quantitative and qualitative data allowed for a richer understanding of treatment experience. The relatively large sample size of young people and inclusion of parents is another strength, given the adolescent voice is particularly missing from day programme research [3] and that parents are an integral part of ITP treatment.

There are several important limitations. The sample size and low response rate, particularly parental response rates, are noteworthy and borderline acceptable [17]. Selection bias can also not be ruled out, due to the study design and use of email surveys. The thematic analysis was also a possible source of bias when the authors (PLB and JB) considered their own role within the analysis process. As working online was also a new experience for the authors, some of their own thoughts about this could have unconsciously influenced theme development.

Perhaps more importantly, the generalisability of the findings beyond the COVID-19 pandemic, is limited. This is due to the aforementioned restrictions on social interaction during the study period, particularly face-to-face interactions, and a high level of uncertainty and anxiety existing within the community. The theme, ‘the best of a bad situation’, is likely the result of these circumstances. Some or part of the themes elicited might not be applicable outside of the pandemic and reported relational limitations of online working may become less relevant. Nevertheless, despite these limitations, useful data about the experience of providing day treatment online was generated that can inform future online day programme treatment developments.

### Future work

The results of this study highlight the need for a specific focus on how to effectively construct the therapeutic relationship in online therapy. Further data is needed to better understand this process. Furthermore, outcome data is needed to determine whether online or hybrid day programme treatment is effective at promoting physical and psychological improvements. Given the convenience and accessibility of online working, larger controlled comparison studies are needed comparing online, or hybrid programmes to face-to-face only programmes.

## Conclusions

This study provides insight into providing day programme treatment online; a new way of working that has been rapidly implemented by many services in response to the COVID-19 pandemic. Overall, the study suggests that using an online platform may offer a more flexible and convenient approach to therapy, especially for parents. Both young people and parents, however, missed the connection of face-to-face working. Going forward, further adaptations might be needed to ensure that the convenience of online therapy does not come at the cost of the therapeutic relationship.

## Data Availability

Data are available from the corresponding author on reasonable request.

## Abbreviations

COVID-19: Coronavirus disease 2019
DP: Day programme
DSM-V: Diagnostic and statistical manual of mental disorders V
FT-AN: Family therapy for anorexia nervosa
FT-ED: Family therapy for eating disorders
IQR: Interquartile range
ITP: Intensive treatment programme
P: Parent
YP: Young person
